# Lipid and glucose profiles in children and adolescents: associations with body composition and metabolic risk factors—a cross-sectional study

**DOI:** 10.1038/s41598-026-53879-5

**Published:** 2026-05-23

**Authors:** Piotr Matłosz, Justyna Wyszyńska, Anna Bartosiewicz, Jarosław Herbert

**Affiliations:** 1 Faculty of Physical Culture Sciences, Medical College, University of Rzeszów, Cicha 2a street, Rzeszow, 35-312 Poland; 2 Faculty of Health Sciences and Psychology, Medical College, University of Rzeszów, Warzywna 1a street, Rzeszow, 35-310 Poland

**Keywords:** Glucose profile, Body composition, Lpid profile, Children, Metabolic risk factors, Cardiology, Risk factors

## Abstract

The increasing prevalence of obesity and metabolic disturbances among children and adolescents underscores the need to evaluate early risk factors. This study aimed to assess the associations between anthropometric indices, physical activity, sleep duration, and lipid and glucose profiles in a sample of Polish youth. A cross-sectional study was conducted among 308 school-aged children and adolescents (7–17 years) in southeastern Poland. Anthropometric parameters and body composition were measured using standardized protocols and bioelectrical impedance analysis. Physical activity and sleep were objectively monitored using ActiGraph accelerometers. Lipid and glucose profiles were obtained via fasting capillary blood samples. A substantial proportion of participants exhibited abnormal lipid levels: 20.8% had borderline or high total cholesterol, 25.6% had elevated triglycerides, and 34.7% had suboptimal high-density lipoprotein cholesterol. Over one-third were classified as overfat or with obesity. Stepwise logistic regression identified low muscle mass as a significant predictor of high triglycerides (OR = 0.20; *p* < 0.001), and higher waist or hip circumference centiles were associated with low high-density lipoprotein levels. Only 25.5% of participants met physical activity recommendations, which was associated with lower triglyceride levels. Sleep duration did not significantly correlate with lipid or glucose outcomes. Dyslipidemia and excess adiposity are prevalent among Polish youth and are strongly associated with unfavorable body composition and low physical activity levels. Waist and hip circumferences emerged as important indicators of lipid risk. These findings highlight the importance of early prevention strategies targeting body composition and physical activity to mitigate long-term cardiometabolic risks.

**Supplementary Information:**

The online version contains supplementary material available at 10.1038/s41598-026-53879-5.

## Introduction

Obesity and metabolic disorders among children and adolescents have become a major public health concern worldwide. Abnormal lipid and glucose profiles in children and adolescents can have serious long-term health consequences^1^. Elevated total cholesterol (TC), triglycerides (TG), and low-density lipoprotein (LDL) cholesterol, combined with reduced high-density lipoprotein (HDL) cholesterol, contribute to early atherosclerosis, arterial stiffness, and hypertension, which may persist into adulthood^2^. Elevated blood pressure (BP) in childhood is increasingly recognized as an important component of early cardiometabolic risk. Studies have shown that elevated BP during childhood is associated with obesity, adverse lipid profiles, and an increased risk of cardiovascular disease later in life. Therefore, assessing BP together with anthropometric indicators and metabolic parameters may provide a more comprehensive evaluation of cardiometabolic risk in pediatric populations^3^. Moreover, children with dyslipidemia and glucose imbalances often present with greater adiposity and hormonal disturbances, which may further contribute to the development of cardiometabolic complications later in life^4^.

The interplay between anthropometric indicators, physical activity levels, and sleep quality is crucial in understanding the determinants of metabolic health in young populations^5^. Several studies suggest that waist-to-height ratio (WHtR), waist-hip ratio (WHR) or waist circumference (WC) are more reliable indicators of central obesity and metabolic risk than body mass index (BMI) alone^6–9^. These parameters allow for better assessment of fat distribution, which is known to be a stronger predictor of cardiovascular and metabolic risk factors^10^. However, while these anthropometric indices provide a practical and non-invasive means to estimate central adiposity, they do not offer direct measurements of body composition. To obtain a more precise evaluation of adiposity and lean body mass, bioelectrical impedance analysis (BIA) has emerged as a widely accepted method for assessing body fat percentage (BFP) in pediatric populations^11^. Unlike BMI, which does not differentiate between lean mass and fat mass, BIA provides a more detailed composition analysis, making it a valuable tool in assessing metabolic and cardiovascular risks in children^12^.

Physical activity-related energy expenditure plays a critical role in the energy balance model, which suggests that obesity is caused by a chronic imbalance in which caloric intake exceeds energy expenditure. This imbalance leads to increased body weight and altered body composition, primarily through the accumulation of fat mass^13^. Reduced physical activity can significantly lower total energy expenditure, resulting in a positive energy balance, subsequent weight gain, and altered cardiovascular health and metabolic function^14^. Beyond caloric intake, suboptimal levels of physical activity, often exacerbated by increased screen time and prolonged sitting, could further aggravate obesity. High screen time, including smartphone use, gaming and watching television, is associated with reduced physical activity and increased sedentary behaviour, both of which contribute to a positive energy balance^15^. Sedentary lifestyles and reduced physical activity levels have been associated with increased obesity rates and cardiometabolic risks in children and adolescents^16,17^.

Unfortunately, a significant proportion of youth fails to meet recommended levels of daily physical activity, leading to concerns regarding their long-term health outcomes^18^. In addition to physical activity, sleep is another essential factor in maintaining a healthy metabolic profile. Poor sleep hygiene, including insufficient sleep duration and lower sleep quality are linked to increased BMI and elevated cardiometabolic risk factors, such as insulin resistance, dyslipidemia, and higher BP in young individuals^19^. Moreover, disruptions in sleep patterns have been associated with increased sedentary behavior and lower engagement in physical activity, further exacerbating metabolic dysfunction^20^.

While previous studies have explored the impact of obesity on lipid and glucose metabolism, there is still a need for a comprehensive understanding of how specific anthropometric and lifestyle factors influence lipid and glucose profiles in children and adolescents. Therefore, this study aimed to evaluate the associations between anthropometric indices (WC, WHR, WHtR, BMI, and BFP), BP, physical activity, and sleep duration with lipid and glucose profiles among children and adolescents. Moreover, this study provides current data on the prevalence of obesity, lipid, and glucose profile abnormalities in a general pediatric population.

## Methods

### Study participants

This cross-sectional study was carried out in 2024 among children and adolescents enrolled in primary and secondary schools in the Podkarpacie region, Poland. A stratified random sampling approach was employed to ensure a balanced representation of both urban and rural areas. Participation was entirely voluntary, with written informed consent obtained from both parents or guardians and the participants prior to data collection. The study was approved by the Ethics Committee (Resolution No. 2/01/2024, dated 17.01.2024) and conducted in accordance with the Declaration of Helsinki.

The number of students enrolled in primary and secondary schools in the Podkarpacie region was approximately 267,300. To determine the appropriate sample size for this study, a statistical calculation was conducted, establishing that a minimum of 288 participants was required to achieve reliable results with a 95% confidence level and a 5% margin of error (taking into account the design effect of def = 0.75 and 0.5 of expected outcome prevalence). To account for potential withdrawals, incomplete measurements, or participant anxiety during examinations, we initially planned to assess 320 children. Ultimately, after applying exclusion criteria and considering cases of withdrawal, the final sample consisted of 308 participants, ensuring sufficient statistical power and representativeness of the study population.

Participants aged 7 to 17 years, enrolled in primary or secondary schools in the Podkarpacie region, Poland, were eligible for inclusion. Written parental consent and participant assent were required. Exclusion criteria included medical contraindications to BIA (e.g. pacemakers, metal implants - none of the original participants were excluded for these reasons) and blood sampling (e.g. bleeding disorders - none, severe needle phobia − 8 participants refused blood sampling), 4 participants were excluded due to technical errors in one or more measurements.

### Anthropometric measurements and body composition

Anthropometric measurements were assessed following The International Society for the Advancement of Kinanthropometry (ISAK) protocol by ISAK Level 3 anthropometrist^21^. Anthropometric assessments included body height, WC and hip circumference (HC). Height was measured to the nearest 0.1 cm using a SECA 213 stadiometer (Seca GmbH & Co. Kg, Hamburg, Germany), with participants standing barefoot in an upright position with head positioned in the Frankfurt plane. WC and HC were measured with a Lufkin steel tape, (Lufkin Rule Company, Apex, North Carolina, USA) at the narrowest point between the lower rib margin and the top of the iliac crest (for WC) and at the level of the greatest protrusion of the buttocks when the subject was standing erect with the feet together (for HC), recorded to the nearest 0.1 cm. WHR and WHtR were calculated to assess central fat distribution.

Abdominal obesity was identified based on Polish WC percentiles, with participants above the 90th percentile classified as having abdominal obesity. Hip circumference was categorized as optimal or high, with high HC defined as values above the 90th percentile, reflecting variations in fat distribution^22^.

Body weight was assessed with a professional body composition analyzer (MC-780 P MA, Tanita Corporation, Tokyo, Japan) with an accuracy of 0.01 kg. Body mass index (BMI) was calculated as weight (kg) divided by height squared (m²). To classify BMI, percentiles were calculated using the OLA/OLAF growth reference for Polish children and adolescents^23^. Additionally, different cut-off points were applied, including OLA/OLAF percentiles, BMI z-score classifications, international standards from the World Health Organization (WHO) and Centers for Disease Control and Prevention (CDC). The CDC define weight status based on percentile cut-offs: underweight: BMI <5th percentile; normal weight: BMI ≥5th and < 85th percentile, overweight: BMI ≥ 85th and < 95th percentile and obesity: BMI ≥ 95th percentile^24^. The following percentile cut-offs were applied to categorize weight status by WHO: underweight: BMI <3rd percentile, normal weight: BMI ≥3rd and < 85th percentile, overweight: BMI ≥ 85th and < 97th percentile and obesity: BMI ≥ 97th percentile^25^. The WHO BMI-for-age Z-score classification was used for categorizing underweight, normal weight, overweight, and obesity in children and adolescents^26^.

Body composition was assessed using BIA with the Tanita MC-780 P MA analyzer (Tanita Corporation, Tokyo, Japan), a validated device for field-based measurements in pediatric populations^27^. All assessments were conducted under standardized conditions, with participants measured in light clothing, barefoot. The analysis was performed in the morning hours, prior to breakfast, to minimize hydration-related variability. The device estimated key body composition metrics, with BFP used for classification. Based on BFP percentile cut-offs, participants were classified into the following categories: underfat: BFP < 2nd percentile; normal: BFP ≥ 2nd percentile and < 85th percentile; overfat: BFP ≥ 85th percentile and < 95th percentile; obesity: BFP ≥ 95th percentile^28^. The other analyzed parameters included muscle mass percentage, fat-free mass percentage (FFM) and total body water percentage (TBW).

### Blood pressure measurement

BP was measured using an automated oscillometric device (Welch Allyn 4200B, Welch Allyn Inc, Aston Abbotts, UK)) following standardized procedures recommended by the European Society of Hypertension^3^. Each participant remained seated at rest for at least 5 min before the measurement. The cuff size was adjusted to fit the participant’s arm circumference. Three consecutive readings of systolic blood pressure (SBP) and diastolic blood pressure (DBP) were taken from the right arm in a quiet environment, with a 1-minute interval between measurements. The average of the two last readings was recorded for analysis. BP status was classified using pediatric percentile charts based on age, sex, and height, according to the centile reference grids according to European Society of Hypertension guidelines for the management of high BP in children and adolescents^3^. Participants were classified as normotensive, high-normal, or hypertensive (stage I or II). Isolated systolic and isolated diastolic hypertension were also identified based on the respective elevation of either SBP or DBP.

### Blood profile

Fasting lipid and glucose levels were assessed using capillary blood samples collected via finger prick in the morning (between 7:00 and 9:00 a.m.), after a minimum 10-hour overnight fast. The procedure was performed by trained nurses following standardized protocols to ensure consistency and participant comfort. The samples were immediately analyzed on-site using the CardioChek^®^ PA Analyzer (PTS Diagnostics, USA), a point-of-care testing device validated for field-based lipid profiling^29^. The WHO includes the CardioChek PA among the recommended dry chemistry devices in its *STEPS Surveillance Manual*, recognizing it as suitable for measuring blood glucose, TC, TG, and HDL cholesterol in large-scale population health assessments^30^. The CardioChek PA provides quantitative measurements of TC, TG, HDL, and fasting blood glucose. LDL was calculated using the Friedewald formula. The device has demonstrated good agreement with standard laboratory-based assays^31^. Lipid results were interpreted based on the pediatric cut-offs, which define acceptable, borderline, and high levels for TC, LDL, and TG (low levels in case of HDL)^32^. Fasting glucose was interpreted using pediatric reference standards for normal and elevated glycemia^33^.

### Physical activity, sedentary and sleep

Physical activity and sleep parameters were assessed using the ActiGraph GT3X-BT Monitor (ActiGraph, Pensacola, FL, USA), worn on the right hip. The monitor was worn continuously for seven consecutive days and nights. Data were collected at 30 Hz and analyzed using ActiLife 6.13 software. Actigraphy is recognized as a valid alternative to polysomnography for sleep assessment^34^ and is widely applied in pediatric populations^35^. Participants wore the device 24 h/day, except during water activities. Parents received detailed instructions regarding proper monitor use. The sleep parameters that were identified using the Sadeh algorithm from the ActiGraphs were: sleep duration (time from when the child fell asleep until they woke up) and WASO (the number of minutes the child was awake between sleep onset and sleep offset)^36^.

After exclusion of the nocturnal sleep episode time non-wear time was defined as ≥ 60 min of continuous zero counts, with a 2-minute allowance for interruptions^37^ The calculation and identification of waking wear time and sedentary time was carried out using the 5-s epoch data. A valid day required ≥ 500 min of waking wear time, and a valid dataset required ≥ 4 days, including at least one weekend day^38^. From valid days, the steps per day and sedentary time (0–100 counts per min.) were extracted^39^.

Based on established public health guidelines, the study population was stratified into subgroups according to adherence to recommended daily step count and sleep duration. In particular, a threshold of ≥ 12,000? steps per day was applied to define compliance with physical activity recommendations in children and adolescents, in accordance with pedometer-based benchmarks proposed in previous research^40^. For sleep, compliance was defined as obtaining 9–11 h? of sleep per night, consistent with National Sleep Foundation recommendations for school-aged children^41^.

### Statistical analysis

The statistical analysis was conducted utilising the SPSS 25 software. The descriptive statistics presented include the mean (M), standard deviation (SD), median (Me) and quartiles 1 and 3 (Q1 and Q3). Categorical variables are described using frequencies and percentages.

The Kolmogorov–Smirnov test was employed to assess the normality of the data. Non-parametric tests were applied directly, without any prior data transformation. Statistically significant differences in the parameters analysed according to gender and age (categories under and above 12 years old) for variables not having normal distributions were assessed with the Mann–Whitney test for continuous variables and the Pearson chi-square or exact Fisher tests for categorical variables. For normally distributed continuous variables, the independent samples t-test was used. The Mann–Whitney test was employed to evaluate the disparities in median lipid and glucose levels across anthropometric, behavioural, and BP dichotomous categories. For parameters with more than two categories, the Kruskal–Wallis test (with chi-square approximation) was used.

A stepwise logistic regression analysis was conducted to identify the most influential factors associated with high TC, high TG, high LDL cholesterol, low HDL cholesterol, and high glucose levels. Independent variables included age (categorized by median), sex, place of residence, anthropometric indicators (BMI, WHR, WHtR, WC, HC, BFP, muscle mass, FFM, TBW), and BP classification. The regression models were built for the total sample as well as separately for boys and girls.

## Results

Table 1 summarizes the participants’ characteristics, presenting age and anthropometric indices, daily physical activity levels, and sleep parameters. Statistically significant gender differences were found for WC, WHR and WHtR. Both WC and WHR was significantly higher in boys compared to girls, suggesting more pronounced central fat distribution in boys. WHtR also showed statistically significant differences, with boys exhibiting slightly higher values than girls (0.45 vs. 0.44). These findings indicate a sex-specific pattern of fat distribution, with boys demonstrating greater central adiposity compared to girls. No significant differences were observed for age, height, weight, sedentary behavior, steps per day, sleep duration, and WASO.

**Table 1:** General characteristics, anthropometric indices, physical activity, and sleep parameters by gender.

Variables	Boys n = 161 Girls n = 147	M	SD	Q1	Me	Q3	p-value
Age (years)	Total	11.5	2.7	9.0	12.0	13.0	
	Boy	11.5	2.8	9.0	12.0	13.0	Z=-0.18; p = 0.857
	Girl	11.4	2.7	9.0	11.0	13.0	
Height (cm)	Total	153.2	16.6	140.2	154.6	165.1	
	Boy	154.9	17.9	140.1	155.3	168.1	Z=-1.578; p = 0.115
	Girl	151.2	14.9	140.3	154.3	163.0	
Weight (kg)	Total	49.0	20.3	32.3	45.2	61.6	
	Boy	51.1	21.2	33.4	48.9	65.2	Z=-1.716; p = 0.086
	Girl	46.8	19.1	31.7	43.5	55.1	
Waist circumference (cm)	Total	68.1	12.9	57.9	65.1	77.8	
	Boy	69.9	13.0	58.4	68.4	79.1	**Z=-2.686; p = 0.007**
	Girl	66.0	12.4	57.4	63.0	73.2	
Hip girth (cm)	Total	85.4	14.9	74.4	84.7	95.7	
	Boy	85.6	14.1	74.7	86.1	96.7	t = 0.179; p = 0.858
	Girl	85.3	15.9	73.2	83.8	92.4	
WHR	Total	0.80	0.1	0.8	0.8	0.8	
	Boy	0.82	0.1	0.8	0.8	0.8	**Z=-6.052; p < 0.001**
	Girl	0.78	0.1	0.7	0.8	0.8	
WHtR	Total	0.44	0.1	0.4	0.4	0.5	
	Boy	0.45	0.1	0.4	0.4	0.5	**Z=-2.58; p = 0.010**
	Girl	0.44	0.1	0.4	0.4	0.5	
Sedentary (h/day)	Total	10.1	2.2	8.5	10.1	11.8	
	Boy	10.2	2.3	8.4	10.3	12.0	Z=-0.506; p = 0.613
	Girl	10.1	2.0	8.5	9.9	11.6	
Steps counts per day	Total	8057.7	3073.7	5782.5	7788.6	10085.1	
	Boy	8298.9	3292.2	5722.1	8027.1	10187.7	Z=-0.915; p = 0.360
	Girl	7780.2	2793.2	5808.7	7632.0	9707.3	
Sleep duration (h/day)	Total	8.3	1.5	7.3	8.3	9.3	
	Boy	8.2	1.5	7.0	8.2	9.2	Z=-1.502; p = 0.133
	Girl	8.5	1.4	7.5	8.5	9.4	
WASO	Total	20.9	8.3	15.3	20.2	25.6	
	Boy	20.9	8.0	15.0	19.7	25.6	Z=-0.316; p = 0.752
	Girl	21.0	8.6	16.2	20.9	25.9	

n – number; M – mean; SD – standard deviation; Q1 – first quartile; Me – median; Q3 – third quartile; WHR – waist-hip ratio; WHtR – waist-to-height ratio; WASO – wake after sleep onset; Z - the result of Mann–Whitney test; t – the result of t-test; p – statistical significance, significant associations are highlighted in bold.

Table 2 shows the distribution of the participants by place of residence, along with gender differences in adherence to recommended levels of physical activity (daily step counts) and sleep duration. The majority of the participants did not meet the recommended number of daily steps (74.5%), with no significant gender differences observed. Less than half of the participants met recommended sleep duration (42.3%), with no significant differences in adherence levels between boys and girls.

**Table 2:** Participants’ residence and adherence to recommended levels of physical activity and sleep by gender.

Variables		Total sample		Boys		Girls		p-value
		N	%	N	%	N	%	
Place of residence	City	200	64.9	101	62.7	99	67.3	χ2 = 0.718; p = 0.397
	Village	108	35.1	60	37.3	48	32.7	
Adherence to daily step count recommendations	Yes	51	25.5	29	27.1	22	23.7	χ2 = 0.311; p = 0.577
	No	149	74.5	78	72.9	71	76.3	
Adherence to sleep duration recommendations	Yes	93	42.3	47	38.8	46	46.5	χ2 = 1.296; p = 0.255
	No	127	57.7	74	61.2	53	53.5	

n – number; χ2- the result of Pearson chi-square test, p – statistical significance.

In Table 3 are presented detailed descriptive statistics for lipid parameters, glucose levels, body composition indices, and BP parameters in the total sample, with comparisons between boys and girls. TC was significantly higher in girls compared to boys (girls: M = 153.5 mg/dL vs. boys: M = 142.6 mg/dL), as was LDL cholesterol (girls: M = 85.4 mg/dL vs. boys: M = 76.9 mg/dL). Girls also exhibited significantly higher BFP (by 4.4%) and mean DBP (girls: M = 69.1 mmHg vs. boys: M = 66.8 mmHg). Conversely, boys had significantly higher muscle mass percentage (boys: M = 76.6% vs. girls: M = 72.5%; ), FFM percentage (boys: M = 80.9% vs. girls: M = 76.5%;), and TBW (boys: M = 59.0% vs. girls: M = 55.8%). The lipid profile, glucose levels, body composition, and BP in the total sample, as well as in boys and girls stratified by age group (< 12 and ≥ 12 years), are presented approximately in Supplementary Tables A.1, A.2, and A.3.

**Table 3:** Lipid profile, glucose, body composition, and blood pressure in the total sample and stratified by gender.

Variables	Boys n = 161 Girls n = 147	M	SD	Q1	Me	Q3	p-value
TC (mg/dL)	Total	147.8	36.4	120.0	146.0	167.0	
	Boy	142.6	40.6	116.0	138.0	160.0	**Z=-4.008;** **p < 0.001**
	Girl	153.5	30.1	136.0	155.0	171.0	
TG (mg/dL)	Total	79.3	40.2	50.0	67.0	91.5	
	Boy	76.7	37.0	50.0	65.0	87.0	Z=-1.058; p = 0.290
	Girl	82.1	43.3	50.0	69.0	95.0	
LDL (mg/dL)	Total	81.0	28.4	61.0	79.0	97.5	
	Boy	76.9	30.7	57.0	71.0	91.0	**Z=-3.799;** **p < 0.001**
	Girl	85.4	25.0	67.0	86.0	104.0	
HDL (mg/dL)	Total	51.6	13.9	43.0	49.0	58.0	
	Boy	51.5	15.8	42.0	48.0	57.0	Z=-1.386; p = 0.166
	Girl	51.8	11.5	44.0	50.0	61.0	
Glucose (mg/dL)	Total	90.2	17.3	84.0	88.0	94.0	
	Boy	91.8	21.6	84.0	90.0	95.0	Z=-1.680; p = 0.093
	Girl	88.5	10.7	83.0	87.0	93.0	
BFP (%)	Total	21.2	8.8	14.1	19.6	27.1	
	Boy	19.1	7.9	12.7	17.6	25.6	**Z=-4.037;** **p < 0.001**
	Girl	23.5	9.2	16.6	20.9	30.5	
Muscle (%)	Total	74.7	8.3	69.2	76.1	81.4	
	Boy	76.6	7.4	70.5	78.1	82.5	**Z=-3.908;** **p < 0.001**
	Girl	72.5	8.7	65.8	75.0	79.1	
FFM (%)	Total	78.8	8.8	72.9	80.4	85.9	
	Boy	80.9	7.9	74.4	82.4	87.3	**Z=-4.037;** **p < 0.001**
	Girl	76.5	9.2	69.5	79.1	83.4	
TBW (%)	Total	57.5	6.6	53.3	58.8	62.8	
	Boy	59.0	5.8	54.2	59.5	63.9	**Z=-3.673;** **p < 0.001**
	Girl	55.8	7.0	50.8	57.9	61.1	
BMI (kg/m2)	Total	20.0	5.1	15.9	18.7	23.7	
	Boy	20.3	4.9	16.1	19.8	24.0	Z=-1.421; p = 0.155
	Girl	19.8	5.4	15.8	18.0	22.4	
Mean SBP (mmHg)	Total	114.0	15.5	103.5	114.0	123.3	
	Boy	114.0	15.5	103.5	114.5	124.0	t = 0.220; p = 0.982
	Girl	114.0	15.5	103.0	113.5	123.0	
Mean DBP (mmHg)	Total	67.9	9.5	61.3	67.5	74.0	
	Boy	66.8	9.3	60.5	66.5	71.5	**Z=-2.214;** **p = 0.027**
	Girl	69.1	9.6	62.5	68.5	75.0	

M – mean; SD – standard deviation; Q1 – first quartile; Me – median; Q3 – third quartile; TC – total cholesterol; TG – triglycerides; LDL – low-density lipoprotein cholesterol; HDL – high-density lipoprotein cholesterol; BFP – body fat percentage; FFM – fat-free mass; TBW – total body water; BMI – body mass index; SBP – systolic blood pressure; DBP – diastolic blood pressure; Z - the result of Mann–Whitney test; t – the result of t-test; p – statistical significance, significant associations are highlighted in bold; TC (mg/dL) Cut-off points: <170 – Acceptable; 170–199 – Borderline; ≥200 – High; TG (mg/dL) Cut-off points for children (0–9 years): <75 – Acceptable; 75–99 – Borderline; ≥100 – High; TG (mg/dL) Cut-off points for adolescents (10–19 years): <90 – Acceptable; 90–129 – Borderline; ≥130 – High; LDL (mg/dL) Cut-off points: <110 – Acceptable; 110–129 – Borderline; ≥130 – High; HDL (mg/dL) ) Cut-off points: >45 – Acceptable; 35–45 – Borderline; <35 – Low; Glucose Cut-off point: <100 – Acceptable; ≥100 - Not acceptable [26, 27].

Table 4 presents the distribution of participants according to lipid profile categories, glucose levels, BFP, BMI interpretations (using OLA/OLAF, z-score, WHO, and CDC criteria), WC, HC and BP categories in the total sample and stratified by gender.

In the total sample, a considerable proportion of participants exhibited abnormal lipid and glucose profiles, indicating potential metabolic risk. 20.8% of participants had TC levels classified as borderline (15.3%) or high (5.5%), while 25.6% had elevated TG, including 9.7% in the high category. LDL cholesterol was also elevated in 13.0% of participants (8.8% borderline and 4.2% high). Additionally, 34.8% of participants had suboptimal HDL cholesterol levels, with 21.8% classified as borderline and 13.0% as low. Abnormal glucose levels were detected in 13.3% of the sample.

In terms of body composition, 34.1% of participants had a BFP above the recommended range (13.3% classified as overfat and 20.8% as with obesity). According to BMI classification (OLA/OLAF centiles), 35.4% of participants had a BMI above the recommended range, including 19.5% classified as overweight (85th–95th percentile) and 15.9% with obesity (3.9% in the 95th–97th percentile and 12.0% above the 97th percentile). Similar trends were observed in alternative BMI classifications, with 12.0% classified as obese using WHO criteria and 15.9% using CDC standards. The BMI z-score analysis further indicated that 36.3% of participants had a BMI above +1SD.

A significant proportion of participants exhibited elevated BP levels, with 33.7% classified as having hypertension-related concerns. This included 13.0% with hypertension, 4.2% with high normal BP, 0.6% with hypertension stage II, 1.9% with isolated diastolic hypertension, and 14.0% with isolated systolic hypertension. Abdominal obesity was observed in 28.6% of participants, while 32.8% had high hip circumference values.

**Table 4:** Categorization of lipid profile, glucose levels, body composition, and blood pressure in the total sample and by gender.

Variables		Total sample		Boys		Girls		p-value
		N	%	N	%	N	%	
TC category	Acceptable	244	79.2	138	85.7	106	72.1	**χ2 = 8.645; ** **p = 0.013**
	Borderline	47	15.3	17	10.6	30	20.4	
	High	17	5.5	6	3.7	11	7.5	
TG category	Acceptable	229	74.4	122	75.8	107	72.8	χ2 = 1.065; p = 0.587
	Borderline	49	15.9	26	16.1	23	15.6	
	High	30	9.7	13	8.1	17	11.6	
LDL category	Acceptable	268	87.0	148	91.9	120	81.6	**χ2 = 10.721;** **p = 0.005**
	Borderline	27	8.8	6	3.7	21	14.3	
	High	13	4.2	7	4.3	6	4.1	
HDL category	Acceptable	201	65.3	96	59.6	105	71.4	**χ2 = 9.214; ** **p = 0.010**
	Borderline	67	21.8	46	28.6	21	14.3	
	Low	40	13.0	19	11.8	21	14.3	
Glucose category	Acceptable	267	86.7	138	85.7	129	87.8	χ2 = 0.277; p = 0.599
	Not acceptable	41	13.3	23	14.3	18	12.2	
BFP category	Underfat	65	21.1	35	21.7	30	20.4	**χ2 = 8.798;** **p = 0.032**
	Normal	138	44.8	62	38.5	76	51.7	
	Overfat	41	13.3	29	18.0	12	8.2	
	Obesity	64	20.8	35	21.7	29	19.7	
BMI percentiles (OLA/OLAF)	< 3 cc	13	4.2	7	4.3	6	4.1	**F = 11.192; ** *p* = 0.044
	3–5 cc	7	2.3	3	1.9	4	2.7	
	5–85 cc	179	58.1	87	54.0	92	62.6	
	85–95 cc	60	19.5	42	26.1	18	12.2	
	95–97 cc	12	3.9	7	4.3	5	3.4	
	> 97 cc	37	12.0	15	9.3	22	15.0	
BMI category (CDC)	Underweight	20	6.5	10	6.2	10	6.8	**χ2 = 10.343;** **p = 0.016**
	Normal	178	57.8	86	53.4	92	62.6	
	Overweight	61	19.8	43	26.7	18	12.2	
	Obesity	49	15.9	22	13.7	27	18.4	
BMI z-score (WHO)	< -2SD	10	3.2	7	4.3	3	2.0	**F = 21.199; ** **p= 0.001**
	-2SD to -1SD	40	13.0	17	10.6	23	15.6	
	-1SD to 0SD	77	25.0	33	20.5	44	29.9	
	0SD to +1SD	69	22.4	37	23.0	32	21.8	
	1SD to +2SD	83	26.9	58	36.0	25	17.0	
	> +2SD	29	9.4	9	5.6	20	13.6	
BMI category (WHO)	Underweight	13	4.2	7	4.3	6	4.1	**F = 11.145; ** **p= 0.010**
	Normal	185	60.1	89	55.3	96	65.3	
	Overweight	73	23.7	50	31.1	23	15.6	
	Obesity	37	12.0	15	9.3	22	15.0	
WC category	Normal	220	71.4	112	69.6	108	73.5	χ2 = 0.574; p = 0.449
	Abdominal obesity	88	28.6	49	30.4	39	26.5	
HC category	Optimal	207	67.2	103	64.0	104	70.7	χ2 = 1.599; p = 0.206
	High	101	32.8	58	36.0	43	29.3	
BP category	Normal	204	66.2	101	62.7	103	70.1	**F = 13.209;** **p = 0.013**
	High normal	13	4.2	8	5.0	5	3.4	
	Hypertension	40	13.0	16	9.9	24	16.3	
	Hypertension stage II	2	0.6	1	0.6	1	0.7	
	IDH	6	1.9	6	3.7	0	0.0	
	ISH	43	14.0	29	18.0	14	9.5	

TC – total cholesterol; TG – triglycerides; LDL – low-density lipoprotein cholesterol; HDL – high-density lipoprotein cholesterol; BFP – body fat percentage; BMI – body mass index; WC – waist circumference; HC – hip circumference; BP – blood pressure; IDH - Isolated Diastolic Hypertension; ISH - Isolated Systolic Hypertension; OLA/OLAF – national BMI reference standards; WHO – World Health Organization BMI classification; CDC – Centers for Disease Control and Prevention BMI classification; χ2- the result of Pearson chi-square test, F -- the result of exact Fisher test; p – statistical significance, significant associations are highlighted in bold; TC (mg/dL) Cut-off points: <170 – Acceptable; 170–199 – Borderline; ≥200 – High; TG (mg/dL) Cut-off points for children (0–9 years): <75 – Acceptable; 75–99 – Borderline; ≥100 – High; TG (mg/dL) Cut-off points for adolescents (10–19 years): <90 – Acceptable; 90–129 – Borderline; ≥130 – High; LDL (mg/dL) Cut-off points: <110 – Acceptable; 110–129 – Borderline; ≥130 – High; HDL (mg/dL) ) Cut-off points: >45 – Acceptable; 35–45 – Borderline; <35 – Low; Glucose Cut-off point: <100 – Acceptable; ≥100 - Not acceptable [26, 27].

Significant gender differences were observed in several metabolic and body composition parameters. Girls had a higher prevalence of borderline or high TC (27.9% vs. 14.3%) and borderline LDL cholesterol (14.3% vs. 3.7%). Borderline or low HDL cholesterol was more frequent in boys (40.4% vs. 28.6%). In body composition, overfat and obesity were more common in boys (39.7% vs. 27.9%). Overweight and obesity (BMI > 85th percentile) were more prevalent in boys across WHO (40.4% vs. 30.6%) and CDC classifications (40.4% vs. 30.6%). Hypertension was more prevalent in girls (16.3% vs. 9.9%), whereas isolated systolic hypertension was more frequent in boys (18.0% vs. 9.5%).

Table 5 presents the associations between TC, TG, LDL cholesterol, HDL cholesterol, and glucose levels with various demographic, anthropometric, and behavioral factors. TG levels were significantly higher in participants with a BMI above the normal range and in those with higher BFP. Post-hoc analysis confirmed significant differences between selected BMI and BFP groups. Similarly, HDL cholesterol varied significantly across BMI, BMI z-score, and BFP categories, with lower values observed in participants with higher adiposity, and post-hoc tests identified specific group differences.

Among BP categories, participants with hypertension stage II had the highest TC (169.0 mg/dL) and LDL cholesterol (102.0 mg/dL), but these differences were not statistically significant. TG were significantly higher in participants with high HC. Adherence to step count recommendations was associated with lower TG levels, whereas adherence to sleep duration recommendations showed no significant differences across lipid or glucose profiles.

**Table 5:** Medians of lipid and glucose levels across anthropometric, behavioral, and blood pressure categories.

Variable		TC (mg/dL)	TG (mg/dL)	LDL (mg/dL)	HDL (mg/dL)	Glucose (mg/dL)
Place of residence	City	146.0	66.0	80.0	49.0	89.0
	Village	147.5	67.0	77.0	49.0	88.0
		Z=-0.704; p = 0.481	Z=-1.194; p = 0.233	Z=-0.782; p = 0.434	Z=-0.662; p = 0.508	Z=-0.437; p = 0.662
BMI category (OLA/OLAF)	< 3 (I)	163.0	63.0	78.0	63.0	90.0
	3–5 (II)	157.0	51.0	88.0	59.0	86.0
	5–85 (III)	147.0	64.0	79.0	50.0	90.0
	85–95 (IV)	143.5	74.5	76.5	46.5	89.0
	95–97 (V)	134.5	68.0	67.0	49.0	92.0
	> 97 (VI)	144.0	79.0	81.0	45.0	85.0
		H = 6.053; p = 0.301	**H = 13.938; p =0.016 †**	H = 2.377; p = 0.795	**H = 24.782; p < 0.001** **I vs. IV p = 0.035** **I vs. VI p = 0.013** **III vs. IV p = 0.028** **III vs. VI p = 0.011**	H = 10.35; *p* = 0.066
BMI category (z-score)	< -2SD (I)	147.5	50.0	73.5	65.0	90.5
	-2SD to -1SD (II)	152.5	53.0	79.5	53.0	86.0
	-1SD to 0SD (III)	147.0	64.0	80.0	51.0	90.0
	0SD to +1SD (IV)	147.0	65.0	80.0	49.0	90.0
	1SD to +2SD (V)	137.0	73.0	71.0	47.0	88.0
	> +2SD (VI)	151.0	81.0	88.0	44.0	85.0
		H = 7.799; p = 0.168	**H = 19.225; p = 0.002** **II vs. VI p = 0.011**	H = 5.154; p = 0.397	**H = 26.451; p < 0.001** **I vs. V p = 0.036** **I vs. VI p = 0.008** **II vs. V p = 0.041** **II vs. VI p = 0.011** **III vs. VI p = 0.020**	H = 10.531; p = 0.062
BMI category (WHO)	Underweight (I)	163.0	63.0	78.0	63.0	90.0
	Normal (II)	147.0	63.0	80.0	50.0	89.0
	Overweight (III)	142.0	73.0	74.0	47.0	90.0
	Obesity (IV)	144.0	79.0	81.0	45.0	85.0
		H = 6.044; p = 0.110	**H = 12.7; p = 0.005** **II vs. IV; p = 0.038**	H = 1.812; p = 0.612	**H = 24.884; p < 0.001** **I vs. III p = 0.014** **I vs. IV p = 0.005** **II vs. III p = 0.004** **II vs. IV p = 0.003**	H = 7.602; *p* = 0.055
WC category	Normal	147.0	64.0	79.0	50.0	90.0
	Abdominal obesity	145.0	73.0	80.0	47.0	86.0
		Z=-0.849; p = 0.396	**Z=-3.31; p = 0.001**	Z=-0.02; p = 0.984	**Z=-3.895; p < 0.001**	Z=-1.73; p = 0.084
HC category	Optimal	147.0	64.0	80.0	50.0	90.0
	High	143.0	72.0	78.0	47.0	86.0
		Z=-1.724; p = 0.085	**Z=-2.54; p = 0.011**	**Z=-0.81; p = 0.418**	**Z=-4.345; p < 0.001**	Z=-1.809; p = 0.070
BFP category	Underfat (I)	149.0	51.0	74.0	55.0	88.0
	Normal (II)	147.0	65.5	80.0	49.0	90.0
	Overfat (III)	142.0	79.0	84.0	47.0	88.0
	Obese (IV)	144.0	73.5	79.0	45.0	86.5
		H = 2.164; p = 0.539	**H = 24.885; p < 0.001** **I vs. III p < 0.001** **I vs. IV p < 0.001**	H = 1.185; p = 0.757	**H = 24.53; p < 0.001** **I vs. III p = 0.006** **I vs. IV p < 0.001** **II vs. IV p = 0.004**	H = 1.934; p = 0.586
BP - category	Normal	146.0	65.5	78.0	49.0	88.0
	High Normal	162.0	68.0	80.0	49.0	87.0
	Hypertension	157.0	73.5	89.0	48.5	93.0
	Hypertension stage II	169.0	89.5	102.0	49.0	85.5
	IDH	139.5	88.5	80.0	43.0	87.5
	ISH	144.0	65.0	76.0	49.0	90.0
		H = 3.725; p = 0.590	H = 2.376; p = 0.795	H = 5.014; p = 0.414	H = 4.932; p = 0.424	H = 8.368; p = 0.137
Adherence to daily step count recommendations	Yes	152.0	50.0	83.0	49.0	87.0
	No	144.0	70.0	74.0	49.0	88.0
		Z=-0.636; p = 0.525	**Z=-3.932; p < 0.001**	Z=-1.466; p = 0.143	Z=-0.396; p = 0.692	Z=-1.093; p = 0.274
Adherence to sleep duration recommendations	Yes	147.0	63.0	80.0	49.0	88.0
	No	144.0	68.0	73.0	49.0	88.0
		Z=-0.824; p = 0.410	Z=-0.67; p = 0.503	Z=-0.953; p = 0.340	Z=-0.525; p = 0.600	Z=-0.455; p = 0.649

Data are presented as median; TC – total cholesterol; TG – triglycerides; LDL – low-density lipoprotein cholesterol; HDL – high-density lipoprotein cholesterol; BMI – body mass index; WC – waist circumference; HC – hip circumference; BFP – body fat percentage; BP – blood pressure; IDH - Isolated Diastolic Hypertension; ISH - Isolated Systolic Hypertension; OLA/OLAF – national BMI reference standards; WHO – World Health Organization BMI classification; Z - the result of Mann–Whitney test; H the result of the Kruskal–Wallis test; p – statistical significance, statistically significant differences are highlighted in bold, † The Dunn’s post hoc test was not statistically significant.

Stepwise logistic regression revealed a significant association between high TG levels and low muscle mass (OR = 0.20; *p* < 0.001). Low HDL cholesterol was significantly associated with higher HC centiles in the total sample (OR = 2.92). Among boys, higher WC centiles were linked to increased odds of low HDL (OR = 3.76). No significant predictors were identified for high glucose levels (Table 6).

**Table 6:** Stepwise logistic regression analysis of risk factors for abnormal lipid and glucose profile.

	Total sample	Boys	Girls
High TC	C	C	BFP_over_obese: OR = 0.13 (0.02–1.16); p = 0.067
High TG	Muscle: OR = 0.20 (0.09–0.47); **p < 0.001**	Muscle: OR = 0.08 (0.02–0.36); **p = 0.001**	C
High LDL	Pleace of residence: OR = 0.16 (0.02–1.22); p = 0.077	age_Me12: OR = 15 (0.02–1.24); p = 0.078	WHR: OR = 0.18 (0.03–1.01); p = 0.052
	WHR: OR = 0.30 (0.09–1.01); p = 0.051	C	C
Low HDL	HC centiles: OR = 2.92 (1.49–5.75); **p = 0.002**	WC_centiles: OR = 3.76 (1.41–10.06); **p = 0.008**	HC_centiles: OR = 2.56 (1.00-6.58); **p = 0.051**
High glucose	C	C	C

Data presented as: OR (CI) – odds ratio with confidence interval; TC – total cholesterol; TG – triglycerides; LDL – low-density lipoprotein cholesterol; HDL – high-density lipoprotein cholesterol; WHR – waist-hip ratio; WC – waist circumference; HC – hip circumference; BFP – body fat percentage; C – constant (reference category in regression analysis). Significant odds ratios are highlighted in bold.

Adherence to step count and sleep duration recommendations was not associated with significant differences in anthropometric or BP variables (Table 7). Although slightly lower values were observed among participants meeting the recommendations, these differences were not statistically significant (all *p* > 0.05).

**Table 7:** Anthropometric and blood pressure variables according to adherence to steps and sleep recommendations.

	Adherence to daily step count recommendations		p	Adherence to sleep duration recommendations		p
	Yes	No		Yes	No	
BMI (kg/m^2^)	17.4	19.2	Z=-1.474; p = 0.140	18.2	19.5	Z=-0.234; p = 0.815
Waist circumference (cm)	60.5	65.7	Z=-1.295; p = 0.195	64.2	67.4	Z=-0.75; p = 0.453
Hip circumference (cm)	77.7	84.9	Z=-1.679; p = 0.093	83.1	87.2	Z=-0.68; p = 0.497
WHtR	0.4	0.4	Z=-0.376; p = 0.707	0.4	0.4	Z=-1.285; p = 0.199
BFP (%)	16.1	20.4	Z=-1.609; p = 0.108	18.9	20.8	Z=-0.849; p = 0.396
Mean SBP (mmHg)	114.5	113	Z=-1.222; p = 0.222	114.5	113.5	Z=-0.448; p = 0.654
Mean DBP (mmHg)	69.0	66.5	Z=-1.641; p = 0.101	68.0	66.5	Z=-1.068; p = 0.286

BMI – body mass index; WHR – waist-hip ratio; BFP – body fat percentage; SBP – systolic blood pressure; DBP – diastolic blood pressure; Z - the result of Mann–Whitney test; p – statistical significance.

Participants living in rural and urban environments did not differ significantly in sedentary time, daily step count, sleep duration, BMI, body fat percentage, systolic or diastolic BP (all *p* > 0.05) (Table 8). A significantly higher WC was observed among participants living in rural areas compared to those from urban areas (*p* = 0.040).

**Table 8:** Sedentary, steps, sleep, anthropometric variables and blood pressure in subjects living in rural and urban environments.

	City	Village	p
Sedentary (h)	9.8	10.5	Z=-1.224; p = 0.221
Steps counts per day	7550.6	8186.7	Z=-0.485; p = 0.627
Sleep duration [h]	8.4	8.2	Z=-0.624; p = 0.533
BMI (kg/m2)	18.1	19.7	Z=-1.842; p = 0.066
Waist circumference (cm)	63.6	68.2	**Z=-2.050; p = 0.040**
Body fat percentage (%)	19.0	20.9	Z=-0.948; p = 0.343
Mean SBP (mmHg)	113.5	114.8	Z=-0.741; p = 0.459
Mean DBP (mmHg)	66.5	68.8	Z=-1.851; p = 0.064

BMI – body mass index; SBP – systolic blood pressure; DBP – diastolic blood pressure; Z - the result of Mann–Whitney test; p – statistical significance. Significant differences are highlighted in bold.

## Discussion

The present study provides a comprehensive assessment of lipid and glucose profiles in relation to anthropometric parameters, physical activity, and sleep among a sample of Polish children and adolescents. Our findings confirm a high prevalence of abnormal lipid profiles, central obesity, and reduced physical activity levels, consistent with global trends in pediatric metabolic health.

Notably, over one-third of the participants presented with BFP values exceeding the healthy range, with 13.3% classified as overfat and 20.8% as with obesity. Similar observations have been reported in studies conducted in younger Polish pediatric populations, where the prevalence of overweight and obesity increases with age already in the preschool period, highlighting the importance of early-life determinants of excess body weight^42^. This is consistent with global trends which found that between 1990 and 2021, the prevalence of obesity in children and adolescents more than tripled worldwide, with projections indicating that by 2050, over 15% of children aged 5–14 years globally will be living with obesity—highlighting a continued and accelerating burden, particularly in younger age groups^43^. Although recent global projections indicate a continued rise in childhood obesity, a systematic review of over 470,000 European children revealed that trends in overweight and obesity have stabilized in most parts of Europe, with the exception of Mediterranean countries, where prevalence has continued to rise—highlighting persistent regional disparities in pediatric weight status across the continent^44^.

A notable finding of this study is the high prevalence of abnormal lipid and glucose levels in the pediatric population. Specifically, 9.7% of participants had high TG, 13.0% had low HDL cholesterol, and 13.3% presented with elevated fasting glucose. These results are consistent with broader international data showing that the prevalence of pediatric dyslipidemia is steadily rising. According to the National Health and Nutrition Examination Survey (NHANES), approximately 20% of individuals aged 12–19 years present with lipid abnormalities^45^. Several studies have confirmed that the prevalence of dyslipidemia varies considerably depending on the country, the specific lipid marker assessed, and the child’s body weight status. Notably, children with obesity demonstrate a significantly higher prevalence, reaching up to 42% in some cohorts^46^. The risk also increases with age: while 15% of children aged 6–11 years exhibit at least one abnormal lipid parameter, this proportion rises to 25% among adolescents aged 12–19 years^47,48^. Moreover, strong evidence supports the concept of lipid tracking from childhood into adulthood, particularly for LDL-C and TC, with pooled correlation coefficients ranging from 0.51 to 0.65, reinforcing the rationale for early detection. These findings suggest that lipid abnormalities identified in childhood often persist, thereby increasing the lifetime risk of atherosclerotic cardiovascular disease^49^.

Our data also support the critical role of body composition, particularly muscle mass and BFP, in shaping lipid outcomes. Stepwise regression identified low muscle mass as a significant predictor of elevated TG levels, particularly among boys, suggesting that poor muscular development may predispose youth to early metabolic disruption. These findings are consistent with a large systematic review of 96 studies across 35 countries, which demonstrated that muscular fitness, especially when assessed through strength and endurance measures, is inversely associated with obesity and clustered cardiometabolic risk factors in children and adolescents. Specifically, muscular endurance was associated with lower obesity in over 68% of reviewed studies and with reduced overall cardiometabolic burden in over 62%. Although the review found limited evidence for a direct association between muscular fitness and lipid markers, our findings provide further support that insufficient muscle mass may contribute to adverse lipid profiles, particularly hypertriglyceridemia, in pediatric populations.

Similarly, a higher HC and WC were independently associated with low HDL cholesterol levels in our cohort, suggesting that regional adiposity plays a critical role in dyslipidemia risk even in children. This aligns with findings by Al-Domi and Al-Shorman (2021), who reported that children with elevated WC showed worse lipid profiles, increased carotid intima-media thickness, and higher levels of inflammatory and endothelial dysfunction markers^50^. Our studies highlights WC as a simple but powerful predictor of early atherosclerosis and metabolic risk in youth.

In our study, only 25.5% of participants met the recommended threshold of ≥ 12,000 steps/day, reflecting a considerable shortfall in daily physical activity among Polish children and adolescents. This aligns with international research showing that most youth globally fail to meet movement behavior guidelines. For instance, among adolescents aged 11–17 years, global surveillance data from 1.6 million individuals revealed that more than 80% were insufficiently active, with consistently lower activity levels in girls and minimal improvement over the past two decades^51^. Our study showed that children meeting step count recommendations had significantly lower TG, highlighting the metabolic benefit of regular physical activity. This aligns with findings by Sohn et al., who reported that children with obesity who engaged in moderate-to-vigorous physical activity (MVPA) had significantly healthier lipid profiles, including lower TG and LDl and higher HDL, compared to their less active peers. These results reinforce the importance of daily movement for improving lipid metabolism, even in the absence of changes in insulin resistance^52^. These findings are further supported by a recent longitudinal study by Agbaje, which showed that higher sedentary time from childhood through young adulthood was associated with worsened lipid profiles. Notably, higher MVPA at age 15 predicted lower LDL at age 24, indicating a lasting protective effect. Although body fat partly mediated these associations, low physical activity demonstrated a more robust lipid-lowering impact, suggesting that even light activity may offer metabolic benefits independent of fat mass^53^.

In contrast, sleep duration showed no significant association with lipid or glucose profiles, despite a substantial proportion (57.7%) of participants not meeting sleep recommendations. These findings may reflect the young age of the sample or the dominance of other metabolic risk factors. However, a large meta-analysis by Che et al., including over 300,000 individuals, demonstrated that both short and long sleep duration were associated with increased risk of metabolic syndrome, following a U-shaped curve. Specifically, short sleep increased the risk of obesity, elevated BP, and impaired glucose regulation, highlighting sleep as a modifiable behavior with broad cardiometabolic implications. Our results suggest that future studies should examine not only duration but also sleep quality and timing to better understand sleep’s role in early metabolic dysregulation^54^.

Although dietary factors were not assessed in the present study, they likely represent important upstream determinants of the cardiometabolic disturbances observed in some participants. Previous research has shown that unhealthy dietary patterns, particularly those characterized by greater consumption of ultra-processed, energy-dense, low-fibre foods, are associated with cardiometabolic alterations and increased cardiometabolic risk in children and adolescents^55^. Inadequate fibre intake has also been linked to less favourable metabolic outcomes and cardiovascular risk markers, underscoring the broader importance of dietary quality for cardiometabolic health^56^. In addition to diet, sedentary behaviour represents another important determinant of pediatric metabolic risk. Systematic reviews indicate that prolonged sedentary time, especially screen-based behaviours, is associated with adiposity and other unfavourable cardiometabolic risk indicators in youth, with some associations observed independently of physical activity levels^57^. These findings suggest that cardiometabolic abnormalities observed in childhood are likely shaped by a complex interaction of behavioural and environmental determinants. Therefore, future studies should examine lipid and glucose abnormalities within a broader behavioural framework that includes dietary quality, fibre intake, sedentary behaviour, and their social and environmental determinants, as these factors are particularly relevant for designing effective prevention strategies and public health interventions. Finally, elevated BP was noted in approximately one-third of the sample, more commonly among boys. Although high BP was not a statistically significant predictor of lipid or glucose abnormalities in this study, its frequent co-occurrence with other metabolic risk factors warrants attention. Previous research by Zhang et al. demonstrated that central adiposity and lipid accumulation indicators, such as WC, BMI, and a novel Relative Children’s Lipid Accumulation Product, were strongly associated with hypertension in youth, with OR exceeding 4. These findings suggest that early BP elevation may reflect underlying lipid storage dysfunction, even when lipid or glucose levels appear within acceptable ranges, and support the need for integrated cardiometabolic screening in pediatric populations^58^.

Taken together, our findings highlight the multifactorial nature of cardiometabolic risk in youth, where body composition, physical inactivity, and central adiposity interact with lipid and glucose abnormalities. This study contributes important epidemiological data from a Central European pediatric population, reinforcing the need for early, multidimensional prevention strategies that integrate body composition monitoring, physical activity promotion, and cardiometabolic screening. These insights may inform public health interventions and policy development aimed at reducing the long-term burden of cardiovascular and metabolic diseases starting in childhood.

The present study has several strengths, including a relatively large sample size, the use of objective methods and validated devices, various interpretation approaches and a comprehensive statistical analysis approach. However, it should be noted that our study is not without its limitations. For instance, due to the cross-sectional nature of the study, it is not possible to make causal inferences about the outcomes. Due to the relatively large sample size and the school-based study design, it was not feasible to include a clinical assessment of pubertal development using the Tanner scale. Similarly, individual-level socioeconomic data were not collected as part of the study protocol. Instead, we included information on place of residence (urban vs. rural), which provides only a limited proxy for environmental and socioeconomic context. We acknowledge that both pubertal status and SES are important factors that may influence body composition, lipid profile, and other cardiometabolic parameters during adolescence. Therefore, the absence of these variables represents a limitation of the present study.

Another limitation is the lack of data on potential diet-related confounders, such as information on subjects’ total caloric intake or the quality of their dietary fat, which could be determinants of both energy balance and serum lipid profile.

Although the accelerometer allows for the objective quantification of overall sedentary time, it does not distinguish between the specific contexts or domains of sedentary behaviour (e.g. screen-based activities versus other forms of sitting). Consequently, variables such as screen time or type of sedentary behaviour were not assessed in this dataset. Further research could be directed towards investigating the potential of domain-specific sedentary behaviours to provide complementary information.

The lack of detailed information on lifestyle-related factors that may influence both lipid metabolism and body composition. In particular, domain-specific sedentary behaviors (e.g., screen time or television viewing), dietary fat quality, and total daily caloric intake were not assessed. These variables are known to affect TG levels and may also contribute to changes in muscle mass. Therefore, the observed association between elevated TG levels and low muscle mass should be interpreted with caution, as residual confounding related to unmeasured behavioral and dietary factors cannot be excluded.

## Conclusions

This study highlights a concerning prevalence of lipid abnormalities and excess adiposity among children and adolescents in southeastern Poland. The findings emphasize the strong association between body composition, particularly low muscle mass and increased WC and HC, and adverse lipid profiles, such as elevated TG and low HDL cholesterol. Physical inactivity further compounded these risks, as participants who did not meet recommended daily step counts were more likely to exhibit unfavorable lipid levels. In contrast, sleep duration was not significantly linked to metabolic outcomes in this cohort, suggesting that other behavioral and physiological factors may play a more dominant role at this developmental stage. The results underscore the importance of early, multifaceted prevention strategies that go beyond BMI to include detailed assessments of body composition and regional fat distribution. Promoting physical activity and monitoring key anthropometric indicators from an early age may be crucial in reducing the long-term burden of cardiovascular and metabolic diseases in the pediatric population.

## Supplementary Information

Below is the link to the electronic supplementary material.

Supplementary Material 1

## Data Availability

The datasets used and/or analysed during the current study are available from the corresponding author on reasonable request.

## Abbreviations

BFP: Body fat percentage
BIA: Bioelectrical impedance analysis
BMI: Body mass index
BP: Blood pressure
CDC: Centers for Disease Control and Prevention
DBP: Diastolic blood pressure
HC: Hip circumference
HDL: High-density lipoprotein cholesterol
ISAK: The International Society for the Advancement of Kinanthropometry
LDL: Low-density lipoprotein cholesterol
M: Mean
Me: Median
MVPA: Moderate-to-vigorous physical activity
Q1: Quartile 1
Q3: Quartile 3
SBP: Systolic blood pressure
SD: Standard deviation
TC: Total cholesterol
TG: Triglycerides
WASO: Wake after sleep onset
WC: Waist circumference
WHO: World Health Organization
WHR: Waist-hip ratio
WHtR: Waist-to-height ratio
