# Supplementary material for: Lipid and glucose profiles in children and adolescents: associations with body composition and metabolic risk factors—a cross-sectional study
Source: Sci Rep. 2026 May 23;16:23575. doi: 10.1038/s41598-026-53879-5 (PMC13421476; doi:10.1038/s41598-026-53879-5)
Supplement: Supplementary file 1 — Supplementary Material 1 [file 41598_2026_53879_MOESM1_ESM.docx]

**Table A.1**. Lipid profile, glucose levels, body composition, and blood pressure in total sample of children and adolescents stratified by age group

| Years | | Age | | | | | p-value |
| --- | --- | --- | --- | --- | --- | --- | --- |
|  |  | M | SD | Q1 | Me | Q3 |  |
| TC (mg/dL) | <12 | 152,1 | 41,3 | 125,5 | 147,0 | 168,5 | Z=-1,569; p=0,117 |
|  | >=12 | 143,6 | 30,4 | 117,5 | 145,0 | 166,0 |  |
| TG (mg/dL) | <12 | 75,9 | 34,3 | 50,0 | 65,5 | 84,0 | Z=-1,139; p=0,255 |
|  | >=12 | 82,5 | 45,0 | 50,5 | 70,0 | 95,5 |  |
| LDL (mg/dL) | <12 | 84,7 | 31,7 | 61,5 | 81,5 | 100,0 | Z=-1,937; p=0,053 |
|  | >=12 | 77,3 | 24,4 | 60,0 | 75,0 | 94,5 |  |
| HDL (mg/dL) | <12 | 52,1 | 14,2 | 42,5 | 49,0 | 59,5 | Z=-0,193; p=0,847 |
|  | >=12 | 51,2 | 13,6 | 43,0 | 49,0 | 57,5 |  |
| Glucose (mg/dL) | <12 | 89,5 | 11,4 | 83,0 | 89,0 | 94,0 | Z=-0,179; p=0,858 |
|  | >=12 | 91,0 | 21,7 | 84,5 | 88,0 | 93,0 |  |
| BFP (%) | <12 | 19,7 | 8,1 | 13,6 | 17,5 | 25,9 | **Z=-3,135; p=0,002** |
|  | >=12 | 22,7 | 9,2 | 15,7 | 22,3 | 28,6 |  |
| Muscle (%) | <12 | 76,0 | 7,6 | 70,3 | 78,2 | 81,6 | **Z=-2,90; p=0,004** |
|  | >=12 | 73,4 | 8,7 | 67,9 | 73,9 | 80,2 |  |
| FFM (%) | <12 | 80,3 | 8,1 | 74,1 | 82,5 | 86,4 | **Z=-3,135; p=0,002** |
|  | >=12 | 77,3 | 9,2 | 71,4 | 77,8 | 84,3 |  |
| TBW (%) | <12 | 58,8 | 5,9 | 54,3 | 60,4 | 63,3 | **Z=-3,570; p<0,001** |
|  | >=12 | 56,2 | 7,0 | 52,3 | 56,2 | 61,2 |  |
| BMI (kg/m2) | <12 | 17,6 | 3,9 | 14,6 | 16,3 | 20,2 | **Z=-8,757; p<0,001** |
|  | >=12 | 22,5 | 5,1 | 18,0 | 22,3 | 26,0 |  |
| SBP (mmHg) | <12 | 108,7 | 14,4 | 98,5 | 108,0 | 119,3 | **t=-6,267; p<0,001** |
|  | >=12 | 119,2 | 14,8 | 110,3 | 118,5 | 128,8 |  |
| DBP (mmHg) | <12 | 65,7 | 9,8 | 59,5 | 64,5 | 70,5 | **Z=-4,465; p<0,001** |
|  | >=12 | 70,1 | 8,8 | 64,0 | 69,5 | 76,0 |  |

M – mean; SD – standard deviation; Q1 – first quartile; Me – median; Q3 – third quartile; TC – total cholesterol; TG – triglycerides; LDL – low-density lipoprotein cholesterol; HDL – high-density lipoprotein cholesterol; BFP – body fat percentage; FFM – fat-free mass; TBW – total body water; BMI – body mass index; SBP – systolic blood pressure; DBP – diastolic blood pressure; statistically significant differences are highlighted in bold; TC (mg/dL) Cut-off points: <170 – Acceptable; 170-199 – Borderline; ≥200 – High; TG (mg/dL) Cut-off points for children (0-9 years): <75 – Acceptable; 75-99 – Borderline; ≥100 – High; TG (mg/dL) Cut-off points for adolescents (10-19 years): <90 – Acceptable; 90-129 – Borderline; ≥130 – High; LDL (mg/dL) Cut-off points: <110 – Acceptable; 110-129 – Borderline; ≥130 – High; HDL (mg/dL) ) Cut-off points: >45 – Acceptable; 35-45 – Borderline; <35 – Low; Glucose Cut-off point: <100 – Acceptable; ≥100 - Not acceptable [26, 27]

**Table A.2**. Lipid profile, glucose levels, body composition, and blood pressure in boys stratified by age group

| Years | | Age | | | | | p-value |
| --- | --- | --- | --- | --- | --- | --- | --- |
|  |  | M | SD | Q1 | Me | Q3 |  |
| TC (mg/dL) | <12 | 151,0 | 50,5 | 122,0 | 142,0 | 165,0 | **Z=-2,156; p=0,031** |
|  | >=12 | 134,7 | 26,5 | 110,0 | 136,0 | 157,0 |  |
| TG (mg/dL) | <12 | 72,8 | 30,9 | 50,0 | 61,0 | 80,0 | Z=-1,138; p=0,255 |
|  | >=12 | 80,4 | 41,8 | 50,0 | 69,0 | 95,0 |  |
| LDL (mg/dL) | <12 | 83,2 | 37,9 | 60,0 | 77,0 | 97,0 | **Z=-2,016; p=0,044** |
|  | >=12 | 70,9 | 20,4 | 53,0 | 69,0 | 85,0 |  |
| HDL (mg/dL) | <12 | 53,0 | 15,3 | 43,0 | 49,0 | 59,0 | Z=-1,794; p=0,073 |
|  | >=12 | 50,0 | 16,1 | 41,0 | 46,0 | 54,0 |  |
| Glucose (mg/dL) | <12 | 91,3 | 11,2 | 84,0 | 91,5 | 95,0 | Z=-0,960; p=0,337 |
|  | >=12 | 92,3 | 28,2 | 84,0 | 88,0 | 94,0 |  |
| BFP (%) | <12 | 18,8 | 8,0 | 12,7 | 16,6 | 25,5 | Z=-0,748; p=0,455 |
|  | >=12 | 19,5 | 7,8 | 12,2 | 20,4 | 26,1 |  |
| Muscle (%) | <12 | 76,7 | 7,4 | 70,5 | 78,8 | 82,4 | Z=-0,446; p=0,655 |
|  | >=12 | 76,5 | 7,4 | 70,2 | 75,4 | 83,2 |  |
| FFM (%) | <12 | 81,2 | 8,0 | 74,5 | 83,4 | 87,3 | Z=-0,748; p=0,455 |
|  | >=12 | 80,5 | 7,8 | 73,9 | 79,6 | 87,8 |  |
| TBW (%) | <12 | 59,4 | 5,8 | 54,5 | 61,2 | 63,8 | Z=-1,184; p=0,236 |
|  | >=12 | 58,5 | 5,8 | 53,8 | 57,4 | 64,2 |  |
| BMI (kg/m2) | <12 | 17,8 | 4,0 | 14,8 | 16,6 | 20,5 | **Z=-6,721; p<0,001** |
|  | >=12 | 22,7 | 4,4 | 19,2 | 23,4 | 25,6 |  |
| SBP (mmHg) | <12 | 108,5 | 14,6 | 100,0 | 108,0 | 120,0 | **t=-4,644; p<0,001** |
|  | >=12 | 119,2 | 14,6 | 109,5 | 118,5 | 129,5 |  |
| DBP (mmHg) | <12 | 65,5 | 9,9 | 59,5 | 64,5 | 70,0 | **Z=-2,006; p=0,045** |
|  | >=12 | 68,0 | 8,7 | 61,5 | 67,5 | 73,5 |  |

M – mean; SD – standard deviation; Q1 – first quartile; Me – median; Q3 – third quartile; TC – total cholesterol; TG – triglycerides; LDL – low-density lipoprotein cholesterol; HDL – high-density lipoprotein cholesterol; BFP – body fat percentage; FFM – fat-free mass; TBW – total body water; BMI – body mass index; SBP – systolic blood pressure; DBP – diastolic blood pressure; statistically significant differences are highlighted in bold; TC (mg/dL) Cut-off points: <170 – Acceptable; 170-199 – Borderline; ≥200 – High; TG (mg/dL) Cut-off points for children (0-9 years): <75 – Acceptable; 75-99 – Borderline; ≥100 – High; TG (mg/dL) Cut-off points for adolescents (10-19 years): <90 – Acceptable; 90-129 – Borderline; ≥130 – High; LDL (mg/dL) Cut-off points: <110 – Acceptable; 110-129 – Borderline; ≥130 – High; HDL (mg/dL) ) Cut-off points: >45 – Acceptable; 35-45 – Borderline; <35 – Low; Glucose Cut-off point: <100 – Acceptable; ≥100 - Not acceptable [26, 27]

**Table A.3**. Lipid profile, glucose levels, body composition, and blood pressure in girls stratified by age group

| Years | | Age | | | | | p-value |
| --- | --- | --- | --- | --- | --- | --- | --- |
|  |  | M | SD | Q1 | Me | Q3 |  |
| TC (mg/dL) | <12 | 153,3 | 29,0 | 136,0 | 155,0 | 170,0 | Z=-0,238; p=0,812 |
|  | >=12 | 153,8 | 31,5 | 137,0 | 153,0 | 174,0 |  |
| TG (mg/dL) | <12 | 79,2 | 37,4 | 50,0 | 66,5 | 95,0 | Z=-0,495; p=0,621 |
|  | >=12 | 85,0 | 48,6 | 52,0 | 71,0 | 98,0 |  |
| LDL (mg/dL) | <12 | 86,4 | 23,6 | 70,0 | 86,5 | 104,0 | Z=-0,362; p=0,717 |
|  | >=12 | 84,5 | 26,6 | 67,0 | 86,0 | 103,0 |  |
| HDL (mg/dL) | <12 | 51,1 | 13,0 | 42,0 | 48,5 | 61,0 | Z=-1,564; p=0,118 |
|  | >=12 | 52,6 | 9,8 | 47,0 | 51,0 | 59,0 |  |
| Glucose (mg/dL) | <12 | 87,5 | 11,3 | 81,0 | 87,0 | 93,0 | Z=-0,65; p=0,516 |
|  | >=12 | 89,5 | 9,9 | 85,0 | 88,0 | 93,0 |  |
| BFP (%) | <12 | 20,7 | 8,2 | 14,5 | 18,1 | 26,5 | **Z=-3,969; p<0,001** |
|  | >=12 | 26,4 | 9,2 | 19,2 | 24,6 | 34,9 |  |
| Muscle (%) | <12 | 75,2 | 7,8 | 69,8 | 77,7 | 81,1 | **Z=-3,892; p<0,001** |
|  | >=12 | 69,9 | 8,8 | 61,7 | 71,5 | 76,6 |  |
| FFM (%) | <12 | 79,3 | 8,2 | 73,5 | 81,9 | 85,5 | **Z=-3,969; p<0,001** |
|  | >=12 | 73,6 | 9,2 | 65,1 | 75,4 | 80,8 |  |
| TBW (%) | <12 | 58,1 | 6,0 | 53,8 | 60,0 | 62,5 | **Z=-4,097; p<0,001** |
|  | >=12 | 53,5 | 7,3 | 47,2 | 55,2 | 59,1 |  |
| BMI (kg/m2) | <12 | 17,4 | 3,8 | 14,5 | 16,0 | 19,5 | **Z=-5,680; p<0,001** |
|  | >=12 | 22,2 | 5,8 | 17,4 | 20,0 | 26,2 |  |
| SBP (mmHg) | <12 | 109,0 | 14,4 | 97,0 | 107,0 | 115,5 | **t=-4,184; p<0,001** |
|  | >=12 | 119,1 | 15,1 | 111,5 | 119,0 | 127,0 |  |
| DBP (mmHg) | <12 | 65,9 | 9,7 | 59,5 | 64,8 | 70,5 | **Z=-4,556; p<0,001** |
|  | >=12 | 72,4 | 8,4 | 67,0 | 71,0 | 78,0 |  |

M – mean; SD – standard deviation; Q1 – first quartile; Me – median; Q3 – third quartile; TC – total cholesterol; TG – triglycerides; LDL – low-density lipoprotein cholesterol; HDL – high-density lipoprotein cholesterol; BFP – body fat percentage; FFM – fat-free mass; TBW – total body water; BMI – body mass index; SBP – systolic blood pressure; DBP – diastolic blood pressure; statistically significant differences are highlighted in bold; TC (mg/dL) Cut-off points: <170 – Acceptable; 170-199 – Borderline; ≥200 – High; TG (mg/dL) Cut-off points for children (0-9 years): <75 – Acceptable; 75-99 – Borderline; ≥100 – High; TG (mg/dL) Cut-off points for adolescents (10-19 years): <90 – Acceptable; 90-129 – Borderline; ≥130 – High; LDL (mg/dL) Cut-off points: <110 – Acceptable; 110-129 – Borderline; ≥130 – High; HDL (mg/dL) ) Cut-off points: >45 – Acceptable; 35-45 – Borderline; <35 – Low; Glucose Cut-off point: <100 – Acceptable; ≥100 - Not acceptable [26, 27]
